# Sonographic Diagnosis of Intraplacental Hematoma in Preterm Pregnancy: A Case Report and Review of Literature

**DOI:** 10.3390/diagnostics16193233

**Published:** 2026-10-07

**Authors:** Sonia-Teodora Luca, Vlăduț Săsăran, Ovidiu Grama, Diana Maria Chiorean, Claudiu Mărginean

**Affiliations:** 1 Doctoral School of Medicine and Pharmacy, “George Emil Palade” University of Medicine, Pharmacy, Science and Technology of Târgu Mureș, Georghe Marinescu Street No. 38, 540142 Târgu Mureș, Romania; sonia.luca@umfst.ro; 2 Department of Obstetrics and Gynecology II, “George Emil Palade” University of Medicine, Pharmacy, Science and Technology of Târgu Mureș, Gheorghe Marinescu Street No. 38, 540142 Târgu Mureș, Romania; 3 Department of Pathophysiology, “George Emil Palade” University of Medicine, Pharmacy, Science and Technology of Târgu Mureș, Gheorghe Marinescu Street No. 38, 540142 Târgu Mureș, Romania

**Keywords:** intraplacental hematoma, preterm pregnancy, sonography, placenta, preeclampsia, placental hematoma

## Abstract

**Background:** Intraplacental hematomas are uncommon placental lesions with potentially significant clinical implications, and their antenatal diagnosis remains challenging. This case report aims to present a rare case of a rapidly enlarging intraplacental hematoma diagnosed antenatally by serial ultrasonographic examinations and to highlight the potential role of ultrasonography in its diagnosis. **Case Presentation:** A 22-year-old gravida 2, para 2 woman presented at 34 weeks and 3 days of gestation for routine antenatal evaluation. Her pregnancy was complicated by intrahepatic cholestasis of pregnancy, treated with ursodeoxycholic acid, with subsequent improvement in biochemical parameters and symptoms. Although asymptomatic, she was noted to have intermittent hypertension. Ultrasonography revealed a well-circumscribed, hypoechoic intraplacental mass measuring 4.37 × 4.0 cm. Color Doppler demonstrated several apparent vascular channels extending through and around the lesion. Repeat ultrasonography 10 min later showed rapid enlargement, raising suspicion of an active intraplacental hematoma. In view of the rapidly progressive sonographic findings and maternal hypertension, emergency cesarean delivery was performed. A newborn was delivered with Apgar scores of 8 and 9 at 1 and 5 min, respectively. Histopathological examination confirmed extensive recent intraplacental hemorrhage, with erythrocyte extravasation into the intervillous spaces and compression of adjacent chorionic villi, without evidence of organization. **Conclusions:** Meticulous placental assessment during prenatal ultrasonography is important, particularly in complicated pregnancies. Color Doppler and serial ultrasonographic examinations may help characterize intraplacental lesions and detect rapid interval changes, facilitating timely obstetric intervention. Histopathological examination remains essential for definitive diagnosis. Further research is needed to clarify the pathogenesis and clinical significance of intraplacental hematomas.

## 1. Introduction and Clinical Significance

The placenta plays a crucial role during pregnancy by mediating the exchange of gases, nutrients, and metabolic waste products between maternal and fetal circulations [1]. Throughout pregnancy, placenta must maintain essential transport, metabolic, and endocrine functions, while undergoing continuous structural and developmental changes [1,2]. In addition, it provides a protective and supportive intrauterine environment, which is essential for normal fetal growth and development [2].

The pathology of the placenta is a well-established contributor to adverse pregnancy outcomes and placental disorders, which encompass a broad spectrum of structural, vascular, and inflammatory abnormalities that may compromise placental function, impair fetal growth, contribute to pregnancy-related complications, and, in severe cases, result in fetal loss [2,3]. These pathological changes primarily include maternal and fetal vascular mal-perfusion, acute or chronic inflammatory lesions, and abnormalities of placental implantation [3].

Intrauterine hematomas are a relatively common complication of pregnancy and may occur at any stage of gestation [4,5,6]. Both early- and late-onset intrauterine hematomas have been associated with an increased risk of adverse pregnancy outcomes, including vaginal bleeding, miscarriage, preterm birth, pregnancy-induced hypertension, preeclampsia, gestational diabetes, fetal growth restriction, and stillbirth [4,5,6,7,8,9]. Despite their reported associations with adverse pregnancy outcomes, the clinical significance of intrauterine hematomas (IUHs) remains controversial [6,7,10,11,12,13]. Since their initial description, studies have reported inconsistent findings regarding their prognostic significance, with some demonstrating an increased risk of adverse perinatal outcomes, whereas others have found no significant association between IUHs and adverse perinatal outcomes [8,9,12,14].

Placental hematomas may be classified according to their anatomical location into four principal types: retroplacental, subchorionic, subamniotic and intraplacental [4]. On ultrasonographic examination, hematomas typically appear as hypoechoic or heterogeneous collections located between the uterine wall and the chorionic membrane [6,15]. Therefore, the sonographic appearance of placental hematomas varies according to their anatomical location. Retroplacental hematomas are located between the basal plate and the underlying myometrium resulting in elevation of the placental parenchyma toward the amniotic cavity. Subchorionic hematomas typically appear as crescent-shaped or triangular hypoechoic or heterogeneous collections situated between the chorionic membrane and the uterine wall. Subamniotic hematomas are generally visualized as oval or cystic masses arising from the fetal surface of the placenta and typically demonstrate no detectable internal vascularity on color Doppler imaging. Intraplacental hematomas, in contrast, are located within the placental parenchyma, predominantly involving the intervillous spaces [4,6,11,12].

Among these, intraplacental hematomas are particularly uncommon, and the available literature remains limited [4,13]. Histopathological studies have demonstrated that rounded intraplacental hematomas exhibit distinctive morphological characteristics compared with parabasal located intervillous thrombohematomas [4,13,16]. An intraplacental hematoma represents a localized accumulation of blood within the placental parenchyma and should be distinguished from other hemorrhagic lesions, particularly intervillous thrombus and rounded intraplacental hematoma [17,18,19]. The Amsterdam Placental Workshop Group Consensus Statement has provided a standardized framework for the classification of placental lesions, which has subsequently been adopted and further discussed in contemporary placental pathology literature [18,20]. According to this Consensus, intervillous thrombus represents a localized accumulation of maternal blood within the intervillous space, frequently associated with fibrin deposition and varying degrees of organization, whereas a rounded intraplacental hematoma is a circumscribed hemorrhagic lesion located within the placental parenchyma [17,18,19]. Although these lesions may demonstrate overlapping macroscopic and ultrasonographic features, their distinction should be based on anatomical localization, morphological characteristics, evolution, and, when available, histopathological findings [17,18].

The objective of this case report is to present a rare case of a rapidly enlarging intra-placental hematoma diagnosed antenatally by serial ultrasonographic examinations and to highlight the potential role of grayscale ultrasonography and color Doppler imaging in its identification and characterization. This case also aims to emphasize the importance of recognizing rapid interval changes in placental lesions, particularly in the context of maternal hypertension, and to discuss the correlation between antenatal imaging findings and definitive histopathological findings.

## 2. Case Report

### 2.1. Clinical Presentation

A 22-year-old woman, gravida 2, para 2, with a spontaneously conceived pregnancy presented to our department for a routine antenatal examination at 34 weeks and 3 days of gestation. She was asymptomatic at presentation and had received routine mineral and vitamin supplementation (including calcium, iron, magnesium, vitamins B1, B2, and B6, folic acid, and vitamin D) supplementation during pregnancy. During the preceding four weeks she had been treated with ursodeoxycholic acid at a dose of 250 mg three times daily (10 mg/kg/day) for symptomatic intrahepatic cholestasis of pregnancy. Her blood group was 0 RhD-positive. She had a previously uncomplicated pregnancy in 2022, when she gave birth to a term baby weighing 2.6 kg, via cesarean section due to breech presentation.

The current pregnancy had been uneventful until 30 weeks of gestation, with no maternal or fetal complications. Serological screening for toxoplasmosis, rubella, cytomegalovirus and herpes simplex virus (TORCH) infections during the first trimester was negative for IgM (immunoglobulin M) antibodies. Immunoglobulin G (IgG) antibodies were positive for all tested infections, indicating previous exposure and/or established immunity. Therefore, repeat serological testing was not considered necessary during the third trimester. Serological testing for human immunodeficiency virus (HIV) types 1 and 2 was performed, with a negative test result. Laboratory investigations, including a complete blood count, renal function tests, coagulation profile, C-reactive protein levels, urinalysis, negative proteinuria and thyroid function tests, were within normal limits. An oral glucose tolerance test was performed at 27 weeks of gestation, which excluded gestational diabetes mellitus.

At 30 weeks of gestation, the patient developed severe pruritus, predominantly affecting the palms of the hands and soles of the feet, with symptoms worsening at night. Laboratory investigations revealed elevated liver enzyme levels, with an alanine aminotransferase (ALT) level of 177 U/L (reference range: 0–34 U/L) and an aspartate aminotransferase (AST) level of 71 U/L (reference range: 11–34 U/L). Total and direct bilirubin levels were 1.4 mg/dL (reference range: 0.3–1.2 mg/dL) and 0.8 mg/dL (reference range: 0–0.5 mg/dL), respectively, while the total serum bile acid concentration was 16 μmol/L (reference range 1–6 μmol/L). These findings were consistent with a diagnosis of intrahepatic cholestasis of pregnancy. As previously mentioned, treatment with ursodeoxycholic acid was initiated, resulting in a significant improvement in the biochemical markers of cholestasis after four weeks of therapy. The patient monitored her blood pressure twice daily, with measurements within the normal range.

At presentation for the routine antenatal examination, a vaginal examination revealed no signs of infection, and the cervix was closed. Cardiotocography showed no uterine contractions, and fetal heart rate variability was within normal limits. There were no clinical symptoms suggestive of preeclampsia, and urine protein testing was negative. However, at admission, the patient was diagnosed with gestational hypertension based on intermittently elevated blood pressure measurements, with a maximum recorded value of 164/100 mmHg. The diagnosis of gestational hypertension was based on new-onset hypertension, in the absence of proteinuria and without clinical or laboratory evidence of maternal end-organ dysfunction.

Ultrasonographic examination revealed an estimated fetal weight of 2591 g. Other sonographic parameters, including amniotic fluid volume, fetal anatomy, cervical length, and fetal Doppler waveforms, were within normal limits. Peripheral and central arterial and venous flow velocities were normal, with no sonographic evidence of fetal anemia or other signs of fetal distress.

Placental ultrasonographic examination was noteworthy. The placenta appeared homogenous without calcifications. However, a well-circumscribed, round, hypoechoic mass measuring approximately 2 cm in diameter was initially identified within the placental parenchyma (Figure 1). Color Doppler imaging demonstrated several large vascular channels extending from the surrounding placental tissue through and around the lesion, suggesting apparent internal vascularity and raising suspicion of an intraplacental hematoma (Figure 2).

A repeated ultrasonographic examination, performed 10 min later, demonstrated a marked increase in the size of the lesion, which had enlarged to 4.37 × 4.0 cm in diameter (Figure 3).

Although intralesional vascularity is not considered a typical feature of hematomas, its presence in this case, together with the progressive enlargement of the lesion on serial ultrasonography, suggested an evolving hemorrhagic process, subsequently confirmed histopathologically. Therefore, these findings should be interpreted in the context of the lesion’s evolution and the overall clinical presentation. All examinations were performed using a Voluson E8 Series BT21 ultrasound system equipped with a C2-9 transducer, using the preset settings for third-trimester obstetric examinations.

In view of the significant ultrasonographic findings, in conjunction with maternal hypertension, the patient was admitted to our department for emergency delivery. An emergency cesarean section was performed, resulting in the delivery of a female baby with Apgar scores of 8 and 9 at 1 and 5 min, respectively. The amniotic fluid was meconium-stained. During surgery, the patient’s blood pressure remained elevated at 170/100 mmHg. Postoperatively, maternal blood pressure remained intermittently elevated, with peak values of 160/110 mmHg. Urinary protein remained negative, and no other clinical or laboratory features suggestive of preeclampsia were identified. The intermittent hypertension was effectively controlled with methyldopa (oral administration of 250 mg twice daily) and magnesium sulfate (intravenous infusion of 20% magnesium sulfate at a rate of 1–2 g/h for 24 h postpartum), and blood pressure subsequently returned to normotensive values once better pain control was achieved. The neonate was admitted to the neonatal intensive care unit and managed as a preterm infant. No abnormalities were identified on subsequent investigations, including cranial and renal ultrasonography and echocardiography.

During hospitalization, the patient received a single 1.5-g intravenous dose of ampicillin/sulbactam for perioperative antibiotic prophylaxis, enoxaparin 0.4 mL subcutaneously once daily for thromboprophylaxis, oxytocin 5 IU intravenously twice daily for three days as uterotonic therapy, as well as treatment for iron-deficiency anemia and concomitant analgesic and anti-inflammatory therapy. Prior to discharge, clinical and ultrasonographic examinations demonstrated a well-involuted uterus, with a 0.3-cm hyperechoic endocavitary collection. The patient’s clinical course was favorable, and she was discharged 8 days after delivery in good general condition. At the time of discharge, she was afebrile, with soft and elastic breasts and established lactation. The cesarean section wound was healing appropriately, blood pressure was stabilized within the normal range, and lochia was serosanguineous. Urination was physiological, and normal bowel function had been restored. Anticoagulation therapy was recommended for an additional 10 days after discharge.

### 2.2. Pathological Findings

The fetal adnexa included a placenta measuring 165 × 115 × 25 mm and weighing 390 g. The maternal surface was mildly lobulated and exhibited a poorly demarcated red–violaceous hemorrhagic area within the placental parenchyma, measuring approximately 45 mm in diameter and consistent with a recent intra-placental hematoma. No macroscopic evidence of placental infarction was identified. The umbilical cord demonstrated marginal insertion and was hypocoiled, measuring 125 × 5 × 5 mm, with the expected three vessels (two arteries and one vein). The amniotic membranes were translucent and attached to the placental margin, with no visible surface deposits.

Microscopically, the placental parenchyma comprised chorionic villi exhibiting a degree of maturation appropriate for the gestational age. An extensive area of recent intraplacental hemorrhage was identified within the placental parenchyma, characterized by extravasation of erythrocytes into the intervillous spaces, with compression of the adjacent villi and no evidence of organization (Figure 4). The villi adjacent to the hematoma demonstrated capillary congestion and mild ischemic changes. The amniochorionic membranes showed no evidence of acute inflammatory infiltrates. The umbilical cord contained three vessels and showed no significant histopathological abnormalities. No evidence of villitis, chorioamnionitis, funisitis, fetal vascular thrombosis, or placental infarction was identified in the examined sections. According to the Amsterdam Consensus, no histopathological features of maternal vascular malperfusion (MVM) were identified, including placental infarction or other characteristic MVM lesions. No features of fetal vascular malperfusion (FVM) were identified, including fetal vascular thrombi or other characteristic FVM lesions.

## 3. Discussion

The pathogenesis of intraplacental hematomas remains incompletely understood and is likely multifactorial [21,22,23]. These lesions are thought to result from disruption of the maternal or fetal placental vasculature, leading to hemorrhage within the placental parenchyma and accumulation of blood within the intervillous spaces [4,13,22,23,24,25]. Several factors can contribute to their development, including maternal hypertensive disorders, placental vascular abnormalities, impaired uteroplacental perfusion, and localized vascular injury [13,21,26,27,28,29]. Intraplacental hematomas may also occur in association with placental abruption or other conditions characterized by abnormal placental perfusion [13,29,30].

Accordingly, mechanisms such as localized disruption of maternal placental vessels or transient alterations in intervillous perfusion may be considered possible but unproven explanations for the hemorrhagic lesion in this case [11,17,22]. These mechanisms should not be regarded as established causal findings, as neither the histopathological examination nor the available clinical data directly demonstrated their occurrence [17,22]. This distinction is consistent with the Amsterdam consensus approach, which emphasizes standardized identification of defined placental lesions while recognizing that their underlying pathophysiological mechanisms may remain uncertain [17,20,22].

The distinctive feature of this case was the sudden onset of maternal hypertension in association with the incidental ultrasonographic detection of an asymptomatic intraplacental hematoma in a preterm pregnancy. Only a limited number of cases of intraplacental hematomas have been reported in the literature, whereas other types of placental hematomas have been more extensively documented. In some cases, these hematomas may be associated with placental abruption and increased maternal, fetal, and neonatal morbidity and mortality.

In the present report, we conducted a comprehensive search of the literature for cases of intraplacental hematoma published up to August 2026, using PubMed and the keyword “intraplacental hematoma.” Only two full-text articles reporting histopathologically confirmed cases of intraplacental hematoma were identified and considered the most relevant publications for the pathological characterization of this rare placental lesion.

Therefore, in 2019, Neville et al. conducted a prospective study of 26 cases of rounded intraplacental hematoma (RIH) and demonstrated that this lesion represents a high-risk placental pathology associated with maternal vascular malperfusion [13]. Compared with placentas exhibiting maternal vascular malperfusion without RIH, those with RIH were associated with a significantly higher incidence of stillbirth and a trend towards an increased risk of placental abruption [13]. The authors highlighted the potential role of antenatal ultrasonography in the detection of abnormal placental lesions, emphasizing the possible clinical significance of RIH when identified during pregnancy [13,17,30].

In 2025, Orlando et al. reported a case of a 25-year-old primigravida at 27 weeks of gestation who presented with severe abdominal pain and mild hypertension. Ultrasonographic examination demonstrated fetal growth restriction and a hypoechoic placental lesion, while subsequent magnetic resonance imaging (MRI) revealed multiple intraplacental lesions suggestive of multifocal hematomas or villous infarcts. The patient subsequently developed preeclampsia with severe features, and histopathological examination of the placenta confirmed multiple infarcts with features of maternal vascular malperfusion [25]. In their article, the authors highlighted the increasing role of magnetic resonance imaging (MRI) as a valuable diagnostic modality in the evaluation of pregnancy-related complications, particularly when ultrasonographic findings are inconclusive or insufficient for definitive diagnosis [25,31]. Placental MRI may further facilitate the characterization of pathological findings associated with preeclampsia-spectrum disorders, including intraplacental hemorrhage, placental infarction, and abnormalities of placental perfusion [21,25,32]. Unlike the case reported by Orlando et al., our case demonstrated an isolated, rapidly expanding intraplacental hemorrhage without clinical symptoms attributable to the hematoma and in contrast, there was insufficient time to perform and interpret MRI before an emergency delivery.

Another important aspect to mention and highlight is the differential diagnosis and imaging pitfalls if a placental hematoma is detected on prenatal ultrasonography, which includes retroplacental hematomas, subchorionic hematomas, subamniotic thrombohematoma, chorioangioma and placental lakes [4,12,33,34,35]. Accurate differentiation among these entities is essential for appropriate clinical assessment and management. Thrombohematomas and chorioangiomas typically appear as heterogeneous echogenic masses on ultrasonography [36,37]. Placental chorioangiomas may mimic hematomas or focal placental infarctions on grayscale ultrasonography and may be differentiated by the presence of internal vascularity on color Doppler examination [38,39]. Placental lakes, defined as anechoic intraplacental spaces measuring at least 1 cm in their largest dimension and demonstrating turbulent blood flow on real-time ultrasonography, are generally considered benign findings and have not been consistently associated with adverse pregnancy outcomes [40,41,42]. There are also several entities that may mimic placental hematomas on prenatal ultrasonography, representing important imaging pitfalls, such as myometrial contractions; however, color Doppler imaging can aid in differentiation, as hematomas typically demonstrate absent or minimal internal vascularity [28]. Although hematomas typically lack internal vascularity, the presence of intralesional Doppler signals may occasionally be observed and should be interpreted in conjunction with the lesion’s evolution, morphology and clinical and pathological findings. Doppler ultrasonography plays an important role in the evaluation of placental abnormalities and may provide valuable information for both diagnosis and clinical management [34]. The following table summarizes the key imaging features distinguishing chorioangioma, placental lake, intervillous thrombus, and subchorionic hematoma (Table 1).

## 4. Conclusions

Intraplacental hematomas are uncommon placental lesions with potentially significant clinical implications and their antenatal diagnosis remains challenging. This case highlights the importance of meticulous placental assessment during prenatal ultrasonography, particularly when a focal intraplacental lesion is identified in the setting of maternal hypertension. Color Doppler and serial ultrasound examinations may provide valuable information regarding lesion characterization and the detection of rapid interval changes, potentially influencing obstetric management.

In the present case, the detection of a rapidly enlarging, asymptomatic intraplacental hematoma in a preterm pregnancy, followed by histopathological confirmation of recent intraplacental hemorrhage, underscores the value of correlating antenatal imaging findings with placental pathology. Although the prognosis of these lesions cannot be established from isolated cases, recognition of intraplacental hematomas may require careful maternal and fetal surveillance and individualized clinical management. Further studies are required to clarify their pathogenesis, sonographic characteristics, relationship with hypertensive disorders, and potential impact on maternal and perinatal outcomes.

## Figures and Tables

**Figure 1:**
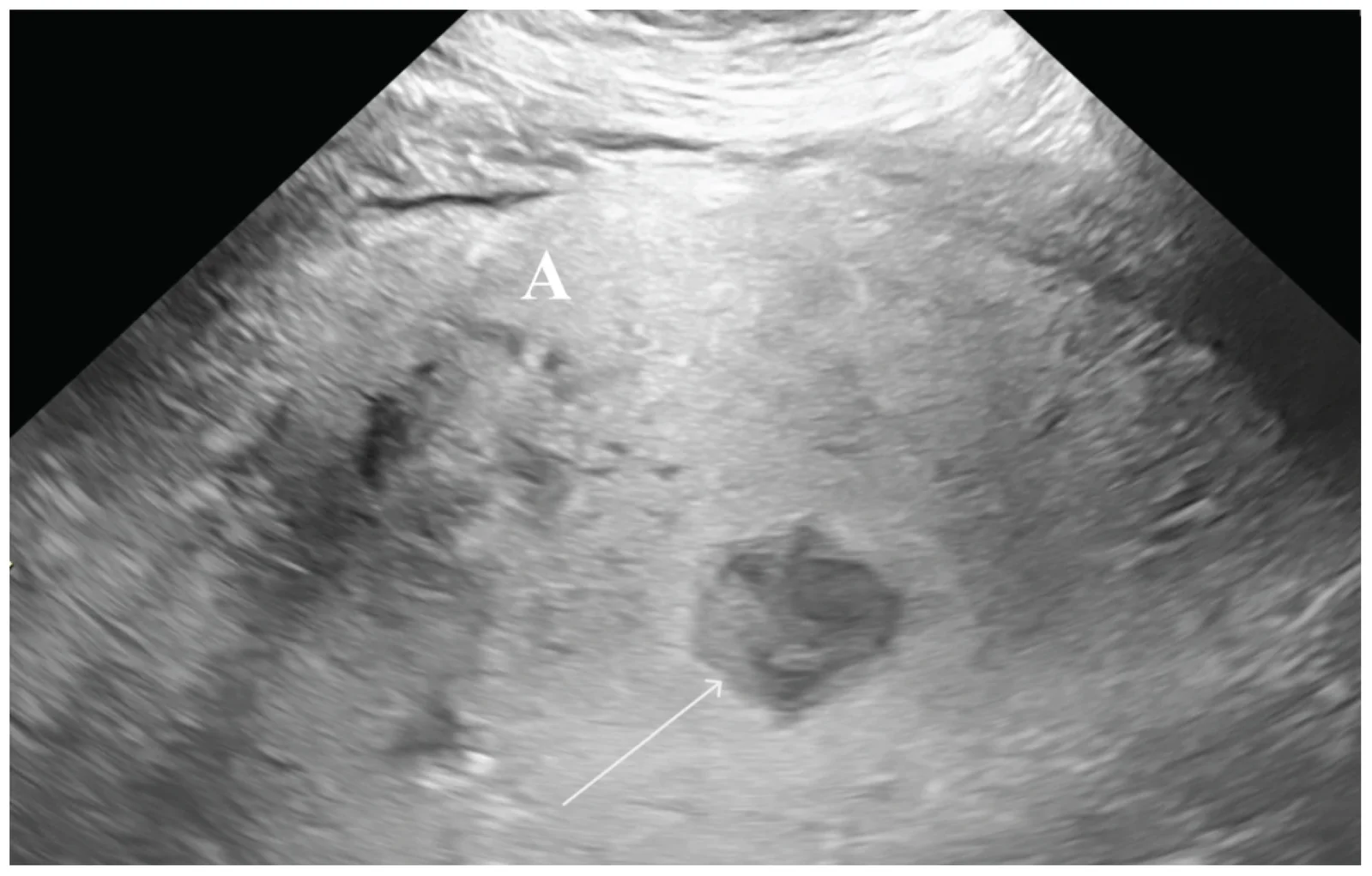
Prenatal ultrasonography of the intraplacental hematoma at 34 weeks and 3 days of gestation: (A)—placental parenchyma, (arrowhead)—hematoma—a well-circumscribed, round, hypoechoic mass within the placental parenchyma.

**Figure 2:**
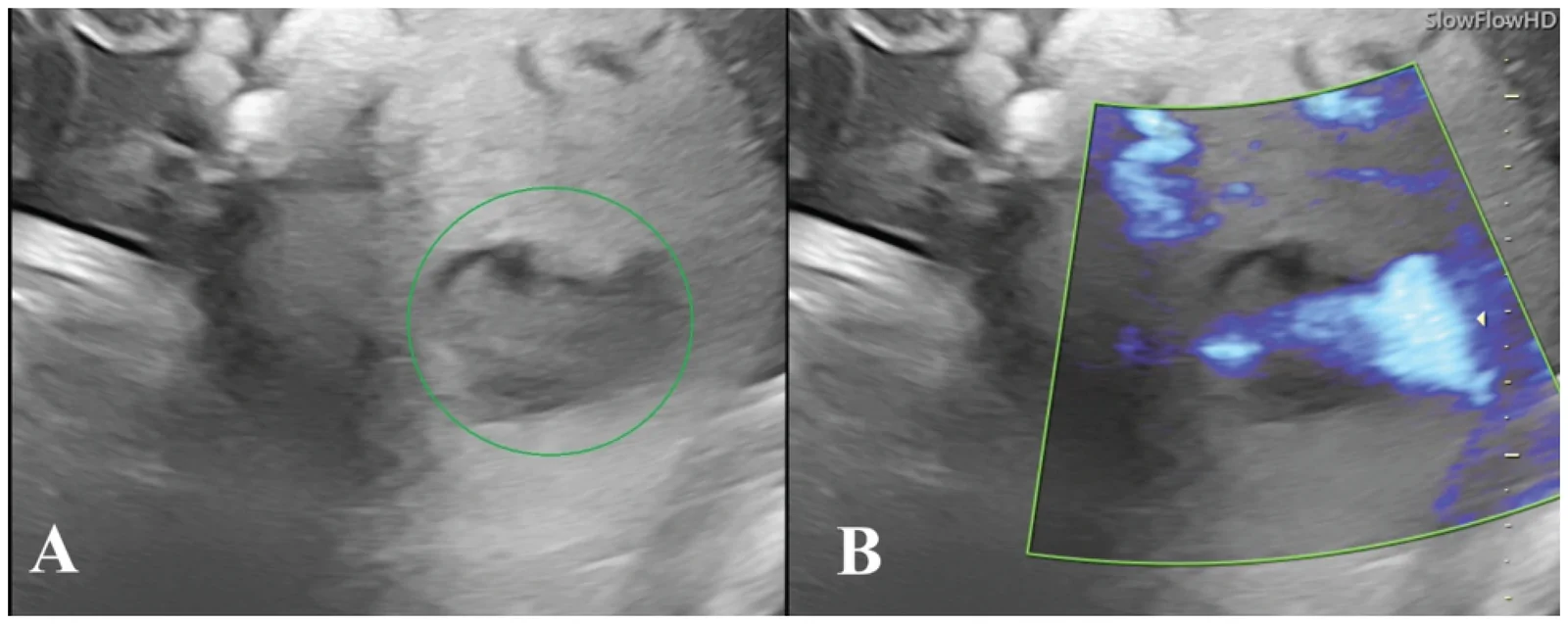
Prenatal ultrasonography of the intraplacental hematoma at 34 weeks and 3 days of gestation: (**A**) placental parenchyma with the hematoma; (**B**) color Doppler images suggesting apparent internal vascularity (SlowFlowHD- software version EC360).

**Figure 3:**
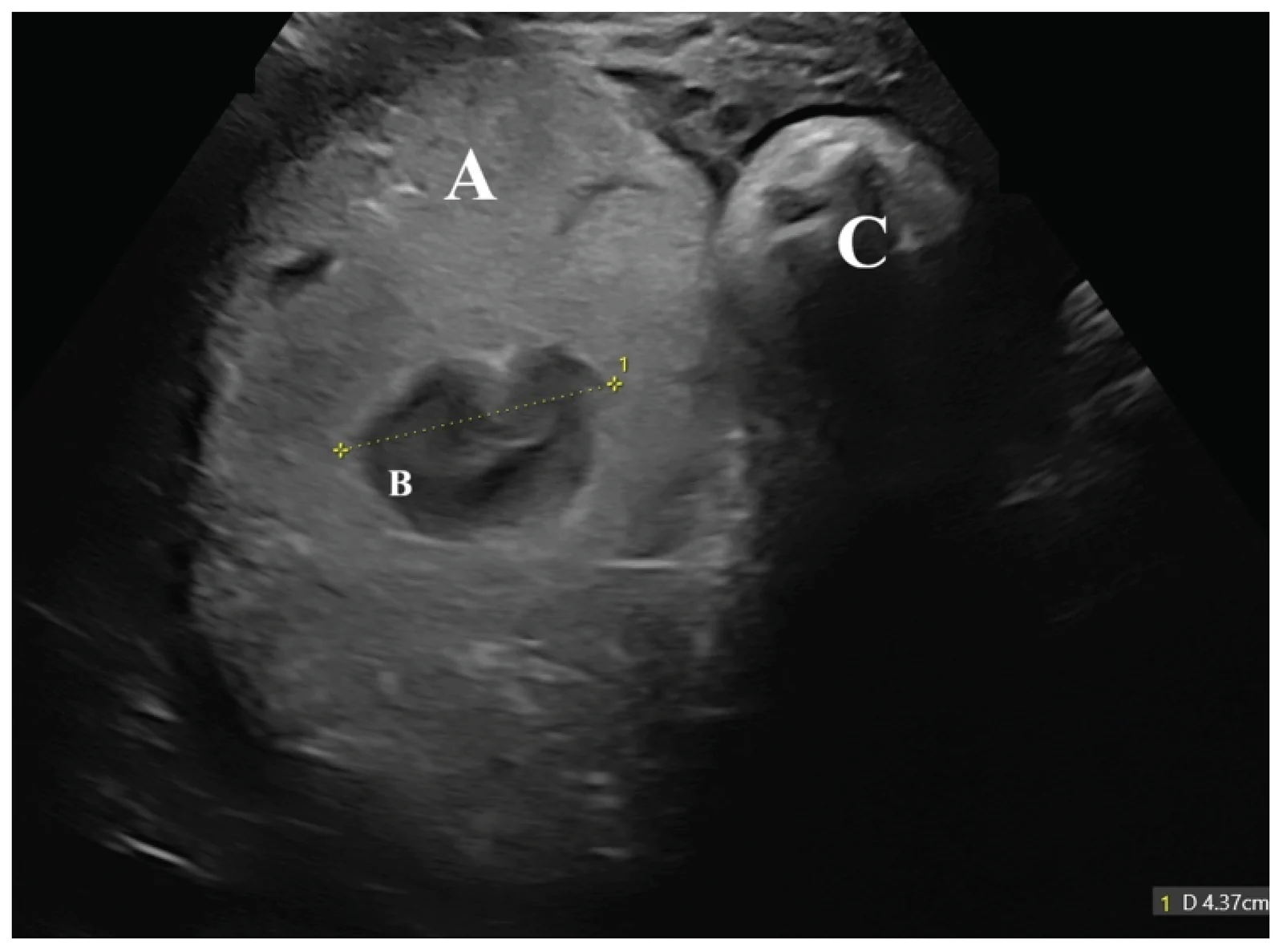
Prenatal ultrasonography of the intraplacental hematoma at 34 weeks and 3 days of gestation: (A) placental parenchyma, (B) intraplacental hematoma, (C) fetal part.

**Figure 4:**
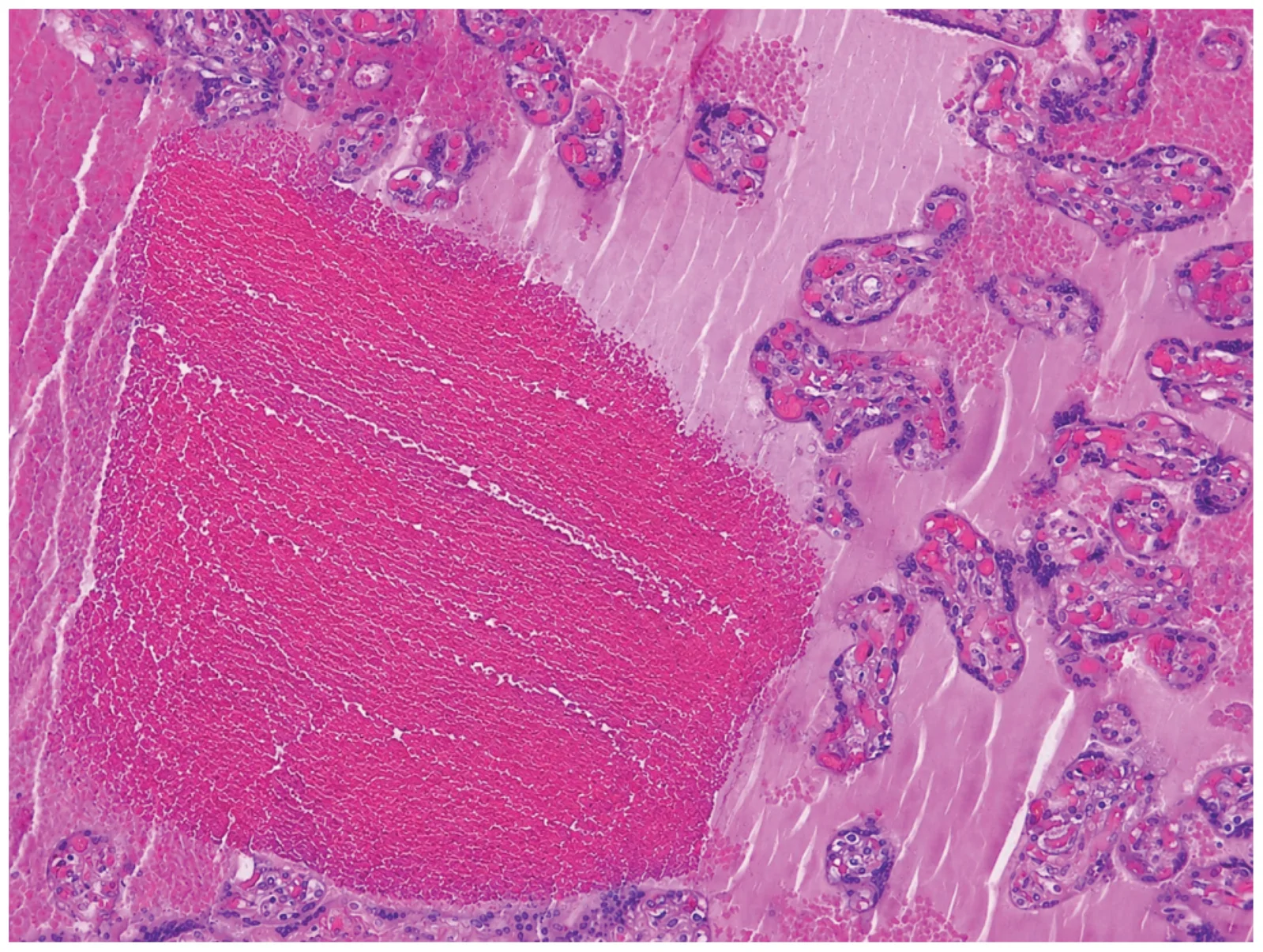
Recent intraplacental hematoma. Hematoxylin and eosin (H&E), 10× magnification. H&E-stained section showing a large intraparenchymal collection of erythrocytes within the placental parenchyma, associated with compression of the adjacent chorionic villi.

**Table 1:** Differential diagnosis for focal intraplacental lesions.

Imaging Feature	Chorioangioma	Placental Lake	Intervillous Thrombus	Subchorionic Hematoma
Pathologic basis	Benign vascular tumor from chorionic mesenchyme [39,43,44,45]	Villous-free intervillous space containing maternal blood [23,41,46,47]	Organized maternal blood/thrombus within the intervillous space, sometimes developing within a placental lake [48,49,50]	Hemorrhagic collection between the chorion and decidual/subchorionic tissues [6,11,15,29]
Location	Usually near the fetal/chorionic surface, frequently close to the cord insertion [39,43,45]	Intraplacental; central/lobular, subchorionic, or marginal [40,41,51]	Intraplacental, frequently involving a pre-existing placental lake [48,49,51]	Usually peripheral/subchorionic, adjacent to the placental margin [6,11,15,29]
Greyscale appearance	Hypoechoic or heterogeneous solid-appearing mass [34,39,43,45,46]	Homogeneous anechoic area surrounded by normal placental tissue [40,41,42,46]	Heterogeneous lesion; increasing echogenicity may reflect organization/thrombosis [46,48,49,51]	Variable echogenicity depending on the age of hemorrhage [6,11,15,46,51]
Color Doppler	Internal vascularity and/or identifiable feeding vessels [34,38,44,45,46]	Usually no high-velocity internal vascularity; slow turbulent/swirl flow may be seen [24,40,41,42,46,51]	No internal blood flow within the thrombosed component [24,46,48,51]	Generally avascular internally [6,11,15,24,46]
Shape	Round or oval, mass-like, usually well circumscribed [34,35,38,43,44,45]	Round/oval or irregular sonolucent space [40,41,42,51]	Variable; may be round/oval with an increasingly echogenic peripheral [48,49]	Crescentic, lentiform, or irregular collection [6,11,15]
Evolution	May enlarge during pregnancy; vascularity may persist [34,35,38,43,44,45]	May increase with advancing gestation [40,41,42]	May develop an echogenic peripheral rim as fibrin and thrombus organize [24,46,48,51]	Changes in echogenicity and morphology as the hemorrhage ages [6,11,15,52,53]
Main characteristics	Solid mass + internal vascularity/feeding vessel [20,34,38,43,44,45,46]	Anechoic maternal-blood space ± slow swirling flow [40,41]	Avascular heterogeneous intraplacental lesion ± echogenic rim [46,48]	Peripheral/subchorionic hemorrhagic collection [6,11,15,52,53,54]

## Data Availability

The original contributions presented in the study are included in the article. Further inquiries can be directed to the corresponding author.

## Abbreviations

The following abbreviations are used in this manuscript:

IUHs	Intrauterine hematomas
TORCH	Serological screening for toxoplasmosis, rubella, cytomegalovirus and herpes simplex virus
IgM	Immunoglobulin M antibodies
IgG	Immunoglobulin G antibodies
HIV	Human immunodeficiency virus
ALT	Alanine aminotransferase
AST	Aspartate aminotransferase
IU	International unit
MVM	Maternal vascular malperfusion
FVM	Fetal vascular malperfusion
RIH	Rounded intraplacental hematoma
MRI	Magnetic resonance imaging
